# Substance Use among Adolescent High School Students in Nigeria and Its Relationship with Psychosocial Factors

**DOI:** 10.34172/jrhs.2020.15

**Published:** 2020-06-06

**Authors:** Adetunji Obadeji, Banji F. Kumolalo, Lateef O. Oluwole, Adedotun S. Ajiboye, Mobolaji U. Dada, Rose Chidindu Ebeyi

**Affiliations:** ^1^Department of Psychiatry, College of Medicine, Ekiti State University, Ado-Ekiti, Ekiti State, Nigeria; ^2^Department of Psychiatry, Ekiti State University Teaching Hospital, Ado-Ekiti, Ekiti State, Nigeria

**Keywords:** Adolescent, Psychological distress, Substance-related disorders, Students

## Abstract

**Background:** Despite the tremendous negative consequences of substances on the health and well-being of adolescents, studies continue to report the high rates of substance use among adolescents. We aimed to identify the pattern of substance use among high school students and its relationship with psychosocial factors.

**Study design:** A cross-sectional study.

**Methods:** The study was conducted in Oct 2019 among students in the senior secondary school in Ado-Ekiti, Ekiti State; southwestern Nigeria. Participants were selected using random sampling, and data were collected using a socio-demographic questionnaire, the Kessler Psychological distress scale and an adapted version of the NIDA-Modified ASSIST. Bivariate analysis and multiple logistic regression were carried out to identify factors associated with psychological distress.

**Results:** Overall, 682 students participated in the study. The lifetime and current prevalence of any substance were 17.3% (95% CI: 14.7%, 20.5%) and 11.7% (95% CI: 9.0, 14.0), respectively. Although most substance use variables increases the risk of psychological distress, history of lifetime substance use AOR= 3.03 (95% CI: 1.19, 7.72,* P* =0.020) and absence of direct parental care AOR=2.04 (1.19, 3.48, P=0.009) significantly increases the risk of experiencing psychological distress. Parental substance use AOR=3.48 (95% CI: 1.57, 7.69, *P* =0.002), male gender AOR=2.97 (95% CI: 1.82, 4.83, P=0.001) significantly increased substance use risk while having married parents AOR=0.50 (95% CI: 0.27, 0.92, P=0.027) and living with parents AOR 0.39 (95% CI: 0.20, 0.75, *P* =0.005) were significant protective factors.

**Conclusion:** The prevalence of substance use among these adolescents was substantial. Drug education initiated in primary school and services aimed at promoting the mental wellbeing of adolescents may go a long way in decreasing substance use among this population.

## Introduction

Adolescence is a challenging stage of life for most individuals and often is characterized by uneven biological, psychological and social development^1^. It often marks the onset of many unhealthy behaviors; including alcohol and drug misuse^2–4^.

Several factors put an adolescent at risk of substance use. The period is characterized by increased adventurous tendencies, peer influences, and risk-taking behaviour^5^. Besides, the adolescent brain is growing, which makes it susceptible to substance use, with alterations in brain structure, function, and neuro-cognition^6–8^. Such neuro-cognitive deficits resulting from alcohol and drug-related neural insults may have harmful effects on subsequent academic, occupational, and social and psychological functioning^8^. Substance use also impacts negatively on families, and communities, with costly social, physical, and mental health consequences^9^.

Despite the tremendous negative consequences of substances on the health and well-being of adolescents, studies continue to report high rates of substance use among adolescents. Different rates and patterns of different substances have been reported depending on the country or region of the country where such a study was carried out^10-14^,.For example, in the US, alcohol was reported as the most commonly used substance; with 72.5% users, followed by cigarettes (46.3%) and marijuana (36.8%)^11^. In Ethiopia on the other hand^2^, the lifetime prevalence of any substance was 65.4% while current substance use among high school students was 47.9%. Alcohol was the commonest drug used with a lifetime and current prevalence of 59% and 40.9% respectively.

In Nigeria, varying rates had been reported. In the Southeast part, for example, the prevalence rate of any substance use among high school was 32.9%, with alcohol being the most commonly used substance (with 29.0% users), and cocaine being the least with 2.1% users^5^. In the southwest parts, from 15.0% to 69.3% adolescents reported to have used any psychoactive substances, more importantly, when local psychoactive substances like kola nut were included ^15–17^. Alcohol remains the most consumed substance followed by cigarette. However, this pattern seems to be changing with tramadol being the second most abused substance^12^.

Several reasons had been reported why adolescents use substances ^4,5,18^. All forms of addictive behaviors including substance misuse had a significant direct relationship with a higher level of psychological distress^19^. The poor drug control policies, with increased substance availability, is another important factor ^4^.

Adolescents are particularly vulnerable to substances, recognizing the mediating role of social and psychological factors may provide further insight into the prevention and treatment of substance-related problems among this population. We aimed to identify the pattern of substance use among high school students and how this is related to socio-demographics and experiencing psychological distress.

## Methods

### Study setting

The study was conducted in Oct 2019 among students in the senior secondary schools in Ado-Ekiti, Ekiti State; southwestern Nigeria. Ado-Ekiti, the capital town of Ekiti State has a population of about Eight hundred thousand people as of 2015. There are 15 public secondary schools in the town, from which the study participants were drawn.

### Sample and Sampling technique

In this cross-sectional survey, participating 5 schools were randomly selected from the public secondary schools considering their geographic locations, i.e. south, north, west, east and central parts of the town. Data were collected from 694 adolescents in the 11^th^ and 12^th^ grade. In each school, a simple random sampling was conducted among students who were present in class each day from senior secondary classes.

### Data collection

All the study instruments were self-administered. After permission from each student, the class teachers, the students were approached in their classes. Before the administration of the questionnaires, the purpose of the study was duly explained to the students and detailed instruction was given on how to complete the questionnaires. Although nearly all the students consented, participants were randomly selected from the students in each class in the Senior Secondary (grades 11^th^ and 12^th^).

### Sociodemographic characteristics

A set of pretested sociodemographic questionnaires was used to elicit sociodemographic characteristics as age, sex, class, religion, family background, history of repeating a class before, number of siblings and parental substance use, and who introduced users to substances. Data on lifetime substance use were collected alongside the sociodemographic variables.

### Psychological distress

Psychological distress was assessed with the 10-item Kessler Psychological Distress Scale (K-10) ^20^. The K-10 scale consists of 10 questions about emotional states, each with a five-level response scale. The measure can be used as a brief screen to identify levels of distress. The tool can be given to interviewees to complete, or the questions can be read to interviewees by the interviewers. Confirmatory factor analysis results show one-dimensional and high internal consistency among adolescents^21,22^.

### Substance use

The lifetime substance use was defined as the use of any under-listed substances in once lifetime either licit or illicit since birth. Current use, on the other hand, was defined as the use of any substance (licit or illicit) in the last four weeks. Substance use was assessed with the DSM-5 Level 2—Substance Use for adolescents, an adapted version of the NIDA-Modified ASSIST^23^. The 15-item instrument assesses different domains of alcohol, tobacco/nicotine, prescription medicine, and illicit substance use in children and adolescents. The instrument was modified to emphasize locally abused substances such as codeine and tramadol. Each item asks the adolescent to rate the severity of his/her use of various substances during the past 4 weeks. The emphasis was on the use and not on the severity of use and so individual items scores were not interpreted for this study. We excluded cultural and socially acceptable substances like kola nut and caffeine.

### Ethical Considerations

Ethical approval was sought from the Research and Ethics Committee of the Ekiti State University Teaching Hospital, Ado-Ekiti (EKSUTH/A67/2019/09/09) and written permission to carry out the study was obtained from the Principal of each respective school. Likewise, written informed consent was sought from each participant via their parents. Students were informed of their right to make a choice either to take part in the study or not. All through the study, confidentiality was ensured.

### Data Analyses

Data analysis was done using Statistical Package for Social Sciences program version 25 (IBM Inc.). Data were presented using frequency distribution tables, bivariate analysis using Pearson Chi-square test (with fisher’s exact or Yate’s correction where applicable) and odd’s ratio (OR) with 95% Confident Interval (CI) were calculated to determine the strength of association between lifetime, current substance use and psychological distress. To adjust for the confounders, factors with a *P* -value of 0.100 or less on bivariate analysis were entered into multiple logistic regression to determine the strength of their association with psychological distress. Adjusted Odds ratios (AORs) were calculated and level of statistical significance was set at *P* <0.05.

## Results

### General measures

Overall, 694 students were enrolled into the study. Of these, 12 sets of questionnaires were adjudged invalid because of inadequate information. Of the 682 analyzed, 26.0% [95% CI: 22.7%, 29.4%] had significant psychological distress; ranging from mild (11.3%), moderate (6.0%) to severe (8.7%). The age ranged from 13-19 yr with a mean of 15.75 Standard Deviation (SD) =1.35. The mean time of initiating any substance was 2.73 yr, and the duration of use range from less than 6 months to a maximum of 10 yr and a mode of 2 years. Of those who were using a substance, friends (62.2%) were reported as the person that introduced them to drugs followed by the parent (16.2%) sibling (10.8%), others (10.9%).

### Socio-demographic characteristics

The majority of the participants were males (57.3%), Christian (86.2%), from a monogamous family background (79.2%), and have their parents still married (89.4%). Thirty-eight of the students (5.6%) reported parental substance use while 67 (9.8%) had repeated a class.

Table 1 shows the pattern of substances used by the participants. The prevalence of lifetime of any substance was 17.3% [95% CI: 14.7, 20.5] and current use of 11.7% [95% CI: 9.0, 14.0]. Alcohol has the highest lifetime and current use, accounting for 13.6% [95% CI: 11.1, 16.4]) and 8.9% [95% CI: 6.9, 11.3] respectively. This was followed by tramadol with lifetime use of 3.8% [95% CI: 2.5, 5.5], current use 2.6% [95% CI: 1.6, 4.1] tobacco with a lifetime use of 3.2% [95% CI: 2.0, 4.8] and current use of 2.5% [95% CI: 1.5, 4.1]. None of the participants reported injection drug use, ecstasy, heroin or cocaine. The lifetime and current use of other substances are shown in Table 1.

**Table 1:** Substance use patterns

**Variables**	**Lifetime use**		**Current use**	
	**Number**	**Percent (95% CI)**	**Number**	**Percent (95% CI)**
Any substance	118	17.3 (14.7, 20.5)	80	11.7 (9.0, 14.0)
Alcohol	93	13.6 (11.1, 16.4)	61	8.9 (6.9,11.3)
Tobacco	22	3.2 (2.0, 4.8)	17	2.5 (1.5, 4.1)
Tramadol	26	3.8 (2.5, 5.5)	18	2.6 (1.6, 4.1)
Cannabis	15	2.2 (1.2, 3.6)	15	2.2 (1.2, 3.6)
Codeine	12	1.8 (0.9, 3.1)	10	1.5 (0.7, 2.7)
Sedatives/hypnotics	9	1.3 (0.6, 2.5)	8	1.2 (0.6, 2.5)

### Relationship between sociodemographic variables and psychological distress

The relationship between sociodemographic variables and the psychological distress is as shown in Table 2. There was no significant relationship between gender, religion and the experience of significant psychological distress. However, having a polygamous family background [OR=1.53 [95% CI:1.03, 2.30)], AOR=1.43 [95% CI: 0.95, 2.165] having a divorced/separated parents COR=1.76 [95% CI:1.05, 2.95], AOR=1.51 [95% CI: 0.89, 2.58], and not living with parents COR=2.21 (95% CI:1.31, 3.75), AOR=2.04 [95% CI:1.19, 3.48] increase the risk of experiencing psychological distress.

**Table 2:** Relationship between sociodemographic and psychological distress

**Variables**	**Psychological distress**				**Crude Odd ratio (95% CI)**	***P*** **-value**	**Adjusted Odd ratio (95% CI)**	***P*** **-value**
	**No**		**Yes**					
	**Number**	**Percent**	**Number**	**Percent**				
Gender								
Male	290	74.5	101	25.5	1.00		-	
Female	215	73.9	76	26.1	1.01 (0.72, 1.44)	0.933	-	-
Religion								
Islam	66	70.2	28	29.8	1.00		-	
Christianity	439	74.7	149	25.3	2.21 (0.77, 2.02)	0.361	-	-
Family background								
Monogamous	411	75.8	131	24.2	1.00		1.00	
Polygamous	94	67.1	46	32.9	1.54 (1.03, 2.30)	0.037	1.43 (0.95, 2.17)	0.086
Parental marital status								
Married	460	75.3	151	24.7	1.00		1.00	
Separated/divorced	45	63.4	26	36.6	1.76 (1.05, 2.95)	0.030	1.51 (0.89, 2.58)	0.127
Live with parents								
Yes	467	75.7	150	24.3	1.00		1.00	
No	38	58.5	27	41.5	2.21 (1.31, 3.75)	0.003	2.04 (1.19, 3.48)	0.009

### Relationship between Substance use and psychological distress

As shown in Table 3, participants with a lifetime history of any substance use COR=2.89 (95% CI: 1.91, 4.38), AOR=3.03 (95% CI: 1.19, 7.72), current history of any substance use COR=3.12 (95% CI: 1.94, 5.01), AOR=1.78 995% CI: 0.66, 4.8450, current use of alcohol COR=3.36 (1.96, 5.74), AOR=1.72 (95% CI: 0.62, 4.81), lifetime use of nicotine COR=2.98 (1.27, 6.99), AOR=1.20 (95% CI: 0.45, 3.15) current tobacco use COR=3.33 (95% CI: 1.64, 8.77), AOR=1.22 (95% CI: 0.38, 3.89), lifetime tramadol use COR=3.00 (1.36, 6.60), AOR 1.25 (95% CI: 0.47, 3.31), current tramadol use COR=3.72 (95% CI: 1.44, 9.58), AOR=1.22 (95% CI: 0.349, 4.25), current cannabis use COR=3.37 (95% CI: 1.20, 9.43), AOR=1.26 (95% CI: 0.36, 4.40), current Codeine use COR=4.40 (95% CI: 1.23, 15.76), AOR=1.35 (95% CI: 0.25, 7.25) were at greater risk of developing psychological distress compared with their counterparts without history such substance use.

**Table 3:** Relationship between Substance use and psychological distress

**Variables**	**Psychological distress**				**Crude Odd** **ratio (95% CI)**	***P*** **value**	**Adjusted Odd** **ratio (95% CI)**	***P*** **value**
	**No**		**Yes**					
	**Number**	**Percent**	**Number**	**Percent**				
Lifetime use of any substance								
No	440	78.0	124	22.0	1.00		1.00	
Yes	65	55.1	53	44.9	2.89 (1.91, 4.38)	0.001	3.03 (1.19, 7.72)	0.020
Current use of any substance								
No	463	77.0	138	23.0	1.00		1.00	
Yes	42	51.9	38	48.1	3.12 (1.94, 5.01)	0.001	1.78 (0.66, 4.85)	0.258
Lifetime Alcohol use								
No	453	76.9	136	23.1	1.00		1.00	
Yes	52	55.9	41	44.1	2.63 (1.67, 4.13)	0.001	0.87 (0.34, 2.18)	0.761
Current Alcohol use								
No	475	76.5	146	23.5	1.00		1.00	
Yes	30	49.2	31	50.8	3.36 (1.96, 5.74)	0.001	1.72 (0.62, 4.81)	0.298
Lifetime Tobacco use								
No	494	74.8	166	25.2	1.00		1.00	
Yes	11	50.0	11	50.0	2.98 (1.27, 6.99)	0.009	1.20 (0.45, 3.15)	0.719
Current Tobacco use								
No	497	74.7	168	25.3	1.00		1.00	
Yes	8	47.1	9	52.9	3.33 (1.64, 8.77)	0.010	1.22 (0.38, 3.89)	0.739
Lifetime Tramadol use								
No	492	75.0	164	25.0	1.00		1.00	
Yes	13	50.0	13	50.0	3.00 (1.36, 6.60)	0.014	1.25 (0.47, 3.31)	0.659
Current Tramadol use								
No	497	74.8	164	25.2	1.00		1.00	
Yes	8	44.4	10	55.6	3.72 (1.44, 9.58)	0.004	1.22 (0.35, 4.25)	0.757
Lifetime Cannabis use								
No	497	74.5	165	25.5	1 .00		1.00	
Yes	8	53.3	7	46.7	2.56 (0.91, 7.16)	0.064	0.85 (0.28, 2.60)	0 .770
Current Cannabis use								
No	498	74.7	169	25.3	1.00		1.00	
Yes	7	46.7	8	53.3	3.37 (1.20, 9.43)	0.014	1.26 (0.36, 4.40)	0.713
Lifetime Codeine use								
No	499	74.5	171	25.5	1.00		1.00	
Yes	6	50.0	6	50.0	2.29 (0.93, 9.17)	0.055	1.10 (0.28, 4.34)	0.890
Current Codeine use								
No	501	74.6	171	25.4	1.00		1.00	
Yes	4	40.0	6	60.0	4.40 (1.23, 15.76)	0.023	1.35 (0.25, 7.25)	0.726
Lifetime use of sedatives								
No	501	74.3	172	25.7	1.00		-	
Yes	5	55.6	4	44.4	2.31 (0.61, 8.71)	0.203	-	-
Current use of sedatives								
No	501	74.6	173	25.4	1.00		-	
Yes	4	50.0	4	50.0	3.64 (0.97, 13.71)	0.215	-	-

### Relationship between lifetime, current use of substances and sociodemographic variables

Table 4 shows the relationship between lifetime, current use of substances and sociodemographic variables. Compared with their female counterparts, the males were significantly at higher risk of both lifetime AOR= 2.97 (1.82, 4.83, *P* =0.001) or current use of any substances AOR= 2.20 (95% CI: 1.28, 3.79, *P* =0.005. Those whose parents use one substance or the other AOR=3.48 (95% CI: 1.57, 7.69, *P* =0.002) were significantly more likely to have a history of lifetime substance use while those whose parents were still married AOR=0.50 (95% CI: 0.27, 0.92, *P* =0.027) who live with their parents AOR 0.39 (95% CI: 0.20, 0.75, *P* =0.005) were less likely to have a history of substance use.

**Table 4:** Relationship between lifetime, current use of substances and sociodemographic variables

**Variables**	**Lifetime substance use**				**Adjusted Odd** **ratio (95%CI)**	***P*** **value**	**Current substance use**				**Adjusted Odd** **ratio (95%C**I)	***P*** **value**
	**No**		**Yes**				**No**		**Yes**			
	**Number**	**Percent**	**Number**	**Percent**			**Number**	**Percent**	**Number**	**Percent**		
Gender												
Female	264	90.7	27	9.3	1.00		270	92.8	21	7.2	1.00	
Male	299	76.5	92	23.5	2.97 (1.82, 4.83)	0.001	329	81.4	62	15.6	2.20 (1.28, 3.79)	0.005
Religion												
Islam	86	91.5	8	8.5	1.00		87	92.6	7	7.4	1.00	
Christianity	477	81.7	111	18.3	2.17 (0.99, 4.75)	0.053	512	87.8	76	12.2	1.49 (0.65, 3.43)	0.349
Family background												
Polygamy	117	83.6	23	16.4	1.00		125	89.3	15	10.7	1.00	
Monogamy	446	82.3	96	17.7	0.86 (0.49, 1.51)	0.609	474	87.5	68	12.5	0.97 (0.51, 1.85)	0.928
Parental Marital status												
Separated/divorced	52	73.2	19	26.8	1.00		60	84.5	11	15.5	1.00	
Married	511	83.6	100	16.4	0.50 (0.27, 0.92)	0.027	539	88.8	76	11.2	0.74 (0 .35, 1.53)	0.410
Parental substance use												
No	442	86.0	72	14.0	1.00		466	90.7	48	9.3	1.00	
Yes	20	52.6	18	47.4	3.48 (1.57, 7.69)	0.002	27	71.1	11	28.9	1.68 (0.71, 3.94)	0.235
Live with parents												
No	48	73.8	17	26.2	1.00		50	76.9	15	23.1	1.00	
Yes	515	83. 5	102	16.5	0.55 (0.29, 1.05)	0.068	549	89.0	68	11.0	0.39 (0.20, 0.75)	0.005

## Discussion

In this study, we looked at the association between psychological distress and substance use among adolescents, and factors associated with these among high school students in a capital town in Nigeria. The lifetime prevalence use of any psychoactive substance among the population study was 17.3%. This is quite low compared to 32.9% in South-eastern Nigeria ^5^or 65.7% ^16^ and 69.3% ^24^ respectively in the south-eastern Nigeria. However, the proportion of students with lifetime use of any substance is relatively higher than 15% in the same south-western part of Nigeria^17^. Similarly, a relatively lower proportion of students in this study was current users of any psychoactive substance. The difference in methodology and extent of psychoactive substance studied may explain these variations. For example, a local psychoactive substance like kola nut, caffeine, and caffeine-containing drugs such as ‘alabunkun’ were included^16^. When compared with similar studies among similar population in developing nations like ours ^2,13,25,26^, the prevalence of substances reported was comarable or even lower, with higher rate reported in Ethiopia, Iraq and most of the articles in a review in Iran^26^. Like in most other studies in this environment ^5,12,16,17^ alcohol was the commonest substances used by the adolescents in this study. The relative availability of alcohol alongside the social and cultural perception of it may explain this. Culture determines to a large extent what constitutes acceptable foods and drinks in many societies. The socio-cultural values and norms of society determine what is socially accepted or rejected^27^. The use of alcohol is more permissible in most cultures, and this may explain its higher prevalence compared to other substances. Besides, advertisement influences social norms about alcohol use ^28^ and contributes to increase drinking among adolescents and young adults ^29^. Against the usual patterns^16,17,30^, tramadol was the second most common substance used by the participants in this study similar to recent findings^12^. This is inconsonant with recent national outcry on the tramadol crisis in Nigeria^31^. The recent increase in the use of prescription opiates such as tramadol and codeine may explain the current pattern noted in this study.

Over a quarter of participants in this study reported significant psychological distress. Except for lifetime alcohol, cannabis, codeine, sedative, and current sedative use, nearly all substance use variables increase the risk of psychological distress. Participants with a life history of substance use were over 3 times more likely to experience significant psychological distress compared with those without such history while participant with current use was about twice more likely to experience psychological distress. This stage of life represents early phase of substance-related disorders (with some at the experimental/initiation stage and few harmful users) and as such there is no marked difference between lifetime users and current users. This may explain the variability in the relationship between lifetime substance use, current substance use and psychological distress.

In a study looking at health risk behaviours among HIV infected adolescents in care^32^, the higher levels of psychological distress were associated with frequent alcohol use and with the risk of having unprotected sexual intercourse^33^. Similarly, a study examining relationship between addictive behaviours and psychological distress among adolescents and emerging adults ^19^ reported all forms of addictive behaviors had significant direct relationship with higher psychological distress ^34^ and those with history of poly drug use were more likely to experience psychological distress, relative to non poly-drug users ^35^.

Besides the history of substance use, family background, parental marital status, and absence of parental care were found to increase the risk of experiencing psychological distress. Those from broken homes, polygamous family and those who do not live with their parents were more at risk of experience psychological distress. Similar observation were reported by other authors ^36–38^. In addition to substance use, family dynamics may predispose adolescents to psychological distress. These same family variables may also predispose adolescents to drug use ^36,38^.

Findings from this study showed that adolescents from married parents, those who live with their parents were less at risk of substance use while and those who reported parental substance use were significantly more likely to present with lifetime history of substance use compared with those who do not have such history^16^. At the same time, those with history of

parental substance use and parental absence were more likely to present with current substance use^39,40^. This is because parental substance use disorders are often characterized by a childrearing environment with poor parenting skills, disadvantaged contexts and adverse childhood experiences ^39^ or direct learning by modelling. These factors may explain why significant proportions of those from a broken home or with a history of parental substance use also reported a higher rate of psychological distress and substance use.

There were some limitations to this study. Firstly, the key variables in the study were self-reported. Data from such may be confounded by social desirability biases, thus limiting acceptability and reliability of the information on the use of various psychoactive substance use. Secondly, substance use or not were not confirmed with biological measures thus narrowing assessment substance use. Those who are using any of the substances but denied such will automatically be excluded. The exposure and outcome were simultaneously assessed, this makes it difficult to ascertain causality between substance use and psychological distress. Despite these limitations, this is one of the few studies exploring adolescents’ substance use and psychological distress in Nigeria and other studies had explored substance use using similar approaches.

## Conclusion

The prevalence of substance use among adolescents studied is relatively low compared to other similar populations of high school students in and outside the country, even when individual substances were considered. On average, most students initiated substance use over two years before the data collection, suggesting preventive strategies are better instituted while in primary school. Besides the association of psychological distress with substance use variables, sociodemographic variables such as being male, being a product of a broken relationship, parental substance use and not living with the parents were significantly associated with substance use. The reason adolescents initiate substance use is multifactorial, therefore, strategies to alleviate youth substance use problems should be broad-based focusing on adolescents’ mental wellbeing, their ability to cope with life challenges, including peer pressures. Other interventions aimed at strengthening family cohesion and ensuring parents serve as a positive role model to their children will go a long way in reducing substance use and misuse.

## Acknowledgements

We will like to appreciate the students, principals, and teachers of those schools that participated in the study for their support during data collection.

## Funding

None.

## Conflicts of interest

None.

## Highlights

- Adolescence is a challenging stage that often marks the onset of many unhealthy behaviors
- Substance use affect psychological health and well-being of adolescents
- Parental substance abuse, marital status and absence of parental care influence both adolescent drug use and psychological health
- Substance use is essentially a problem of adolescent’s boys, therefore more attention needs to be paid on this sub-population
